# Notes and News

**Published:** 1898-04-23

**Authors:** 


### Notes and News.

(Collected and Communicated.)

### HOSPITAL MEETINGS.

London Lock Hospital.—Annual Meeting, Thursday, April 28th, 4.30, at the Hospital.

City of London Hospital for Diseases of the Chest.—The Duke of Connaught will preside at the Dinner, in aid of this institution, to be held at the Hotel Cecil on May 12th.

A Hindoo, charged with setting fire to the Plague Hospital at Bombay, has been sentenced to transportation for life.

A special appeal is being made for £2,000 to clear the debt on the Mary Wardell Convalescent Home. Miss Wardell will have a stall on behalf of her home at the united sale of work at the Albert Hall on April 28th and 29th.

One of the most interesting spectacles at the Easter volunteer musters was that afforded by the London companies of the Volunteer Medical Staff Corps at Walmer, by whom a most complete display of bandaging and stretcher drill was given.

The arrangements for the wounded in the Soudan campaign appear to be working excellently, though the heat is naturally very trying to the sick. The boats in which they are floated down the Nile to the base hospital are provided with every possible comfort.

A generous and anonymous donor has presented some railway stock, valued at £11,000, to the endowment fund of the Blackburn Infirmary, and, in addition, has presented a sum of £2,000 towards the Victoria wing, which was erected in commemoration of the Diamond Jubilee.

A new steam laundry has been decided upon for Addenbrooke's Hospital at Cambridge. The matron accompanied two members of the committee to London, where she studied the method of dealing with the linen, including the disinfection. The committee has reduced the contract price of tea for hospital use from 2s. a pound to 1s. 9d. Two shillings a pound for tea is higher than the majority of middle-class mortals can afford to pay.

Fifty thousand pounds have been left to Glasgow charities by the late Mr. Adam Teacher, of that city.

The wounded soldiers in the West African operations have hitherto suffered from the disadvantage of a tedious and injurious journey from the scene of warfare to the permanent hospital at Freetown, Sierra Leone. To obviate this a hospital equipment has been sent to the front, including a complete field hospital, which will doubtless be heartily welcomed by the wounded.

The Princess of Wales has sent the following touching letter to the little cripples belonging to a class in connection with the Ragged School Union and Shaftesbury Society, Ratcliff Highway, who had sent 3s. 9½d. as a subscription to the Prince of Wales's Hospital Fund: "Miss Knollys is desired by the Princess of Wales to thank the little crippled children very much for their kind contributions to the Prince of Wales's Hospital Fund, and to assure them that their small offerings are more highly appreciated by their Royal Highnesses than the most costly gifts of those less afflicted than themselves. The Princess is sure that the little cripples will have great satisfaction in feeling that by their own self-denial they are sending help to their fellow-sufferers."

At the opening of the new fever hospital at Worcester, Earl Beauchamp spoke of the special advantage to the town of such a building, since a penalty of £10 was enforced on manufacturers who allowed any portion of their goods to enter a house where a case of infectious disease was existent. In a place like Worcester that was a particular hardship, for in many of the cottages isolation was impossible, and in the gloving industry probably the whole living of a household depended on that source, and the whole means of livelihood was taken away when there was a case of infectious disease in the house. A novelty was introduced into the opening ceremony by the planting by various visitors of trees to adorn the grounds. The Mayor planted a black pear tree, Lord Beauchamp an acer, while a scarlet oak, a Worcestershire sack apple, and many other trees will in the future make the surrounding grounds ornamental.

The new rules as to hospital stoppages will throw upon the army medical officers a responsibility which, according to the manner in which it is exercised, will conduce much to their popularity or otherwise in the barrack-room. The old rule has been that any man in hospital, not suffering from injuries received on duty, has been put on hospital stoppages of 7d. a day. In future, however, half of this sum will be remitted on the certificate of the doctor that the man's sickness has been contracted as the direct result of military service, and under such circumstances as to be beyond his own control. Here indeed is a problem in etiology. When a man is laid up with a cold, a pleurisy, or an attack of rheumatism, after a hard field day, or after sentry duty in bad weather, is the doctor to allow his medical imagination to wander back to possible causes, and give Tommy Atkins the benefit of the doubt; or is he sternly to refuse to certify to what he cannot be sure of, and so give the ratepayer the benefit of the fact that it is impossible in many cases to certify why a man has become ill.

